# Role of Gene Methylation in Antitumor Immune Response: Implication for Tumor Progression

**DOI:** 10.3390/cancers3021672

**Published:** 2011-03-29

**Authors:** Alfonso Serrano, Isabel Castro-Vega, Maximino Redondo

**Affiliations:** 1 Department of Immunology, Hospital Clinico Universitario, Campus Universitario Teatinos S/N, 29010 Malaga, Spain; 2 Department of Biochemistry, CIBER ESP, Hospital Costa del Sol, Marbella, Málaga, Carretera de Cadiz km 187, 29603, Spain

**Keywords:** methylation, tumor, HLA, clusterin

## Abstract

Cancer immunosurveillance theory has emphasized the role of escape mechanisms in tumor growth. In this respect, a very important factor is the molecular characterization of the mechanisms by which tumor cells evade immune recognition and destruction. Among the many escape mechanisms identified, alterations in classical and non-classical HLA (Human Leucocyte Antigens) class I and class II expression by tumor cells are of particular interest. In addition to the importance of HLA molecules, tumor-associated antigens and accessory/co-stimulatory molecules are also involved in immune recognition. The loss of HLA class I antigen expression and of co-stimulatory molecules can occur at genetic, transcriptional and post-transcriptional levels. Epigenetic defects are involved in at least some mechanisms that preclude mounting a successful host-antitumor response involving the HLA system, tumor-associated antigens, and accessory/co-stimulatory molecules. This review summarizes our current understanding of the role of methylation in the regulation of molecules involved in the tumor immune response.

## 1. DNA Methylation as a Tumoral Epigenetic Phenomenon

An estimated 3–5% of the cytosine residues in genomic DNA are methylated and exist as 5-methylcytosine (m5C) [1]. The genomic distribution of the latter is not random; it is clustered in specific regions. Furthermore, DNA methylation patterns are cell type specific. Cytosine methylation occurs after the S-phase and is catalyzed by DNA methyltransferase (DNMT); S-adenosyl-methionine (SAM) is the methyl donor in this reaction. Cell type specific DNA methylation occurs through both template mediated mechanisms and *de novo* methylation reactions [2,3]. DNMT1 uses hemimethylated DNA as a preferential template [4], whereas DNMT3a and DNMT3b may methylate, hemimethylate and unmethylate DNA with equal efficiency, suggesting that DNMT3a/3b function as *de novo* methylases [3]. DNMT2 has been shown to methylate integrated retroviral sequences [5]. Genomic DNA methylation may occur through the enzymatic demethylation of DNA (“active” demethylation) as well as from a failure to maintain methylation after the S-phase (“passive” demethylation) [6-8]. In murine and bovine zygotes, even before DNA replication, active demethylation of paternally inherited DNA occurs, through unknown mechanisms [9].

Epigenetics describes an inheritable alteration in gene expression that is independent of a change in the DNA sequence. Ample experimental evidence suggests that cancer is both a genetic and an epigenetic disease. Along with genetics, epigenetics may add further explanation of the complexity of abnormal changes that are produced in cancer cells. The first described epigenetic changes in human cancer included losses of DNA methylation [10]. Such an alteration of DNA methylation throughout the genome has been observed in various cancers affecting a wide variety of tissues [11]. It is apparent that metastases are even more susceptible to cancer-linked DNA hypomethylation than are primary tumors. It is also apparent that a more extensive genomic hypomethylation is more frequently observed in metastases than in primary tumors. Many subsequent reports have confirmed the frequent occurrence of overall genomic hypomethylation in cancers, in relation to control tissues [12].

It is important to note that the hypomethylation of genomic DNA repeats [13,14] is largely responsible for the global DNA hypomethylation that is so frequently observed in cancers [15]. Promoter hypermethylation has been noted in tumor suppressor genes, while homeodomain genes are commonly found in the cancer genome [16].

Paradoxically, despite prevalent increases in global DNA methylation, overall deficiencies in the m5C content of DNA are found in almost every type of neoplasm [17,18]. For example, a murine model of prostate cancer displays both satellite DNA hypomethylation and gene locus-specific hypermethylation in the tumors [19].

Just as there are cancer-type specific differences in DNA hypermethylation patterns [20], so some DNA sequences are more or less hypomethylated, depending on the kind of cancer [21]. Seminoma displays an especially large amount of genomic hypomethylation, although, in this case, genomic hypomethylation may be a reflection of the unusually hypomethylated DNA present in the cell of origin [22,23]. Moreover, for some DNA sequences, cancer-linked DNA hypermethylation can be seen in some specimens and hypomethylation in others. Cancer-linked hypo- and hyper-methylation are generally independent processes, although many studies show both genomic changes occurring concurrently in the same neoplasm.

Global DNA hypo- and hypermethylation of 55 genetic loci have been observed in diverse ovarian epithelial tumors and normal somatic tissues [24]. Thus, DNA hypo- and hypermethylated genetic loci usually co-exist in the same tumor. Moreover, progressive aberrations in promoter hypermethylation, the hypomethylation of DNA repeats, or global DNA hypomethylation, can be seen in comparisons of premalignant lesions and malignant neoplasms, as well as in *in vitro* and *in vivo* models of tumor progression [25]. Cross-talk between demethylation and *de novo* methylation pathways during tumorigenesis has been suggested. In this respect, methylation-demethylation has been viewed as a process of physiological compensation for the inappropriate methylation of CpG islands overlapping promoters of tumor suppressor genes. This suggests that DNA hypomethylation might occur early in oncogenesis and be followed by hypermethylation [26] as a kind of increased, compensatory *de novo* methylation consequent to the genomic hypomethylation. However, analysis of the association between malignancy-linked DNA hyper- and hypomethylation, using quantitative measures of hypermethylation at gene loci, global DNA hypomethylation and semi-quantitative data on satellite DNA hypomethylation in various ovarian epithelial carcinomas, has shown that hypo- and hypermethylation are not interdependent. Accordingly, hypomethylation cannot be described as a mere consequence of DNA hypermethylation, and *vice versa* [24]. In summary, both DNA hyper- and hypomethylation are linked to carcinogenesis and present inter-relationships other than that of one being dependent on the other. They may have one or more steps in common in the (still) poorly understood pathways that produce these divergent epigenetic changes [27].

## 2. The Regulation of Methylation as an Epiphenomenon is Indissolubly Linked to Changes in Chromatin Structure

Nucleosomes, the fundamental building blocks of eukaryotic chromatin, consist of 147 bp of DNA wrapped 1.6 times around an octamer composed of two H3-H4 histone dimers bridged together as a stable tetramer that is flanked by two separate H2A-H2B dimers. The addition of other factors, such as linker histone H1 and non-histone chromatin proteins, results in higher order chromatin organization and compaction [28]. These core histone molecules are among the most evolutionarily conserved proteins, highlighting the likelihood of critical functions being performed by these small proteins [29]. Chromatin, rather than being a passive platform for storing genetic information, can regulate transcriptional processes through post-synthetic modifications of its main components: DNA and histones. Although DNA methylation and histone modification are carried out by different chemical reactions and require different sets of enzymes, there seems to be a biological relationship between the two systems that plays a part in modulating gene repression programming in the organism. DNA methylation and specific histone modifications influence each other during mammalian development and also during the aberrant gene expression patterns observed in cancer. It seems that the relationship can work in both directions: histone modifications can help to direct DNA methylation patterns, and DNA methylation might serve as a template for some histone modifications after DNA replication. These connections might be accomplished through direct interactions between histones and DNA methyltransferases. Differential methylation during mammalian development is established through two counteracting mechanisms: a wave of indiscriminate *de novo* methylation and a mechanism ensuring that CpG islands remain unmethylated. Recent studies strongly suggest that the establishment of the basic DNA methylation profile during early development might be mediated through histone modification. Histones are currently known to be subjected to nine different types of posttranslational modifications, although methylation and acetylation have been found more frequently as epigenetic marks which are maintained with high fidelity through cell division. There is a relationship between methylation and acetylation, since some of the lysine residues that are methylated in histones H3 and H4 are also found to be substrates for acetylation. Transcription seems to be turned off directly through the interaction of repressor molecules with promoters [30,31]. This is followed by the transcription factor dependent recruitment of a complex that contains the histone methyltransferase G9a and enzymes with a histone deacetylase activity. Deacetylation resets the lysine residues so that G9a can catalyze methylation of the histone. Histone methylation may serve as a marker of transcriptionally active euchromatin or of transcriptionally repressed heterochromatin. *De novo* DNA methylation is carried out by the DNA methylatransferase enzymes DNMT3A and DNMT3B complexed with DNMT3L, a closely related homologue that lacks methylatransferase activity [32-34]. DNMT3L recruits the methylatransferases to DNA by binding to histone H3 in the nucleosome, but contact between DNMT3L and the nucleosome is inhibited by all forms of methylation on H3K4. According to this model, the pattern of methylation of H3K4 across the genome might be formed in the embryo before *de novo* DNA methylation. H3K4 methylation might be directed by sequence-directed binding of RNA polymerase II, which recruits specific H3K4 methylatransferases. As RNA polymerase II is bound mostly to CpG islands in the early embryo, only these regions are marked by H3K4me, whereas the rest of the genome is packaged with nucleosomes containing unmethylated H3K4. This understanding of the relationship between DNA methylation and certain histone modifications also provides insight into the aberrant gene expression patterns observed in cancer. Preliminary evidence suggests that some cancer cells express an abnormally high concentration of methylatransferases [35]. It seems that a large number of CpG islands can become *de novo* methylated at an early stage of tumorogenesis [36]. As many of these methylation events occur at the promoter of genes that are already repressed in the normal tissue before transformation [37], *de novo* methylation profile in tumors is not formed as a result of selection.

## 3. HLA Antigen Changes in Malignant Cells

The revival of the cancer immunosurveillance theory has emphasized the role of escape mechanisms in tumor growth [38]. As a result, tumor immunologists have been focusing their investigations on the identification and molecular characterization of the mechanisms by which tumor cells evade immune recognition and destruction [39,40]. Among the many escape mechanisms identified, alterations in classical and non-classical HLA class I and class II expression by tumor cells are of particular interest to tumor immunologists and clinical oncologists because of the critical role they play in the generation of tumor antigen (TA)-specific immune response, as well as their ability to modulate the interaction of natural killer (NK) cell and T cell populations [41].

The human leukocyte antigen (HLA) class I antigens, HLA-A, -B and -C, which form the class I major histocompatibility complex in humans, take part in the recognition of virally infected, grafted or transformed cells by cytotoxic T cells [42]. The HLA genes are located on chromosome 6p21 and are expressed in most somatic tissues. Selective loss of expression of these loci has frequently been observed in human tumors [43,44]. The expression of class I antigens has been studied by immunohistochemistry (IHC) in different tumors in correlation with clinicopathological characteristics. Reduced expression has been observed in kidney, prostate, stomach, colon and germ cell testicular cancers and has been associated with tumor invasiveness and aggressiveness [43]. Loss of HLA class I antigen expression can occur at the genetic, transcriptional and post-transcriptional levels [45].

HLA class II molecules in humans play a key function in triggering the adaptive immune response by presenting antigenic peptides to CD4+ T helper cells. HLA class II molecules are expressed constitutively in dendritic cells, B cells and cells of the monocyte/macrophage lineage. Moreover, cytokines, mainly INFgamma, may further increase, or *de novo* induce, HLA class II in macrophage-like cells or in cells of extrahematopoietic origin [46]. Expression of the HLA class II gene is mainly regulated at the transcriptional level. Triggering of the HLA class II transcriptional activator is mediated by the non-DNA-binding specific co-activator CIITA, which is required for both constitutive and inducible expression [47]. Given its role in the homeostasis of immune response, much attention has also been focused on the expression of HLA class II in disease states, such as cancer, in which the immune response appears to be hampered. In tumor cells, HLA class II expression is known to be associated with a better prognosis for colorectal cancer and breast cancer [48,49]. Moreover, in ovarian carcinoma and lymphoma, a positive correlation between tumor-infiltrating lymphocytes and the amount of HLA class II in tumor cells has been documented. Conversely, HLA class II expression has been linked to disease progression and poor prognosis in melanoma [50-52].

## 4. Methylation Regulation as an Epigenetic Modification in the Alteration of HLA Expression and of Associated Molecules

The notion that epigenetic alterations modulate HLA changes in malignant cells was first suggested by studies showing that HLA expression can be recovered by treatment with DNA methylation and HDCA inhibitors [53,54]. In this respect it has been demonstrated that epigenetic defects are involved in at least some mechanisms that preclude mounting a successful host-antitumor response, involving the HLA system, tumor associated antigens, and accessory/co-stimulatory molecules [55,56]. DNA methylation participates in regulating the expression of HLA class I antigens (A, B and C), which are CpG-rich at their gene promoters [57]. It has been shown that down-regulation of HLA class I in esophageal carcinoma is a common mechanism for transcriptional inactivation, caused primarily by DNA hypermethylation [58], as well as in melanoma, where 5-aza-2′-deoxycitidine significantly enhances the constitutive expression of HLA class-I. In this respect, it has been shown that in the MSR3-mel melanoma cell line the methylation status of HLA-A and HLA-B genes is higher than in the autologous PBL genome [59]. Moreover, changes in HLA class I expression may also reflect alterations in the transcriptional regulation of antigen processing machinery components. These play a critical role in the assembly and presentation of functional HLA class I antigens [60]. In this regard, with the exception of rare examples of TAP1, tapasin and LMP subunit mutations found in some small cell lung carcinoma, cervical carcinoma, neuroblastoma and cutaneous melanoma cell lines, structural alteration in the antigen processing machinery component genes appears to be a rare event. Methylation of the tapasin and/or TAP2 promoter has been reported in melanoma and RCC cell lines and treatment with DNA demethylation agent 5-aza-2′-deoxycytidine (5-AC) results, not only in the enhancement and/or reconstitution of tapasin and TAP2, but also in TAP1 transcription and translation. In addition, treatment of some esophageal squamous cell carcinoma, colon carcinoma, RCC and melanoma cell lines with 5-AC and/or HDCA results in the induction of Antigen Processing Machinery (APM) component transcription and translation [61,62]. In the same context, hypoacetylation of the H3 histone leads to deficient expression of TAP-1, a critical APM component, and ultimately to reduced HLA class I expression in a lung carcinoma cell line [60]. Similar studies have also demonstrated that specific APM components, including LMP7, TAP-1, TAP2, and tapasin, can be epigenetically regulated in certain tumors [63].

The genes encoding HLA class II antigens, as well as those encoding the accessory molecules involved in HLA class II presentation, are coordinated regulated and their expression, at the transcriptional level, is primarily controlled by the class II transactivator protein (CIITA). Four promoters regulate different isoforms of CIITA expression in a cell-specific manner. Constitutive CIITA expression in dendritic cells (DCs) is controlled by promoter I (D-CIITA), while CIITA in B-lymphocytes is driven by promoter III (B-CIITA) [64]. Epigenetic mechanisms have also been found to underlie defects in HLA class II expression in malignant cells. Both hypermethylation of the CIITA promoter IV [65] and/or modification of chromatin structure by histone deacetylation may result in defective CIITA expression in tumor cells, causing a loss of INF-gamma-inducible HLA-DR expression [64]. It is thought that CpG dinucleotide methylation of CIITA promoter IV DNA, as well as histone deacetylation, severely impair recruitment of transcription factors such as IRF-1, Stat-1 and USF-1 to CIITA-PIV, thereby reducing CIITA transcription [66].

## 5. Many of the Antigens Present in the HLA Context in Tumor Cells are the Result of Epigenetic Modifications

A group of genes with germline-specific expression may become activated in a wide variety of tumors. Such genes are called cancer-germline (CG) or cancer testis. Among these genes, MAGE-A1 was the first initially discovered in a melanoma that encodes two antigens recognized by cytolytic T lymphocytes [67]. In one study, expression of the MAGE-A1 gene was detected in approximately half of all melanomas and in other tumors of different histological origins, but was absent in normal adult tissue with the exception of the testis [68]. MAGE-A1 belongs to a family of at least 12 closely-related genes located in the terminal portion of the long arm of the x chromosome [69]. Because the sequence of the MAGE-A1 gene in melanoma cells is identical to that in normal blood cells [67], the gene has to be reactivated in tumors [70]. Both the GAGE and the LAGE gene families form part of this group of germline-specific genes [71]. It has been shown that promoter methylation plays a fundamental role in reactivation of these genes; treatment with 5-AC can induce gene expression in some tumor cell lines that are negative for this gene [72]. It has also been shown that the B′B region of the MAGE-A1 gene promoter is methylated in different cell lines that are negative for expressing this gene [73]. Furthermore, there is a hypothesis that the expression of CG genes may be a hallmark of stem cells and linked to stem cell biology [74]. Corroboration for this was provided in a previous study reporting the expression of several CG genes in undifferentiated human mesenchymal stem cells, but not in their differentiated derivatives [75]. It could be that CG gene expression in tumors does not result from a gene activation process, but reflects the expansion of constitutively expressing cancer stem cells. However, it seems that for some CG genes, such as certain members of the MAGE, the GAGE or the LAGE gene families, their expression in somatic tumors does not reflect the expansion of expressing precursor stem cells, but results from an epigenetic activation process that occurs during tumorigenesis [76]. These genes are usually silent in normal tissues, with the exception of the testes, which are inaccessible to the immune system, and so their specific proteins are tolerated. This would explain the lack of tolerance, and consequent recognition by cytotoxic T lymphocytes [67,77]. As remarked above, methylation deregulation during tumorigenesis leads to the ectopic expression of different types of proteins that can interfere with tumor behavior.

## 6. Epigenetics Directly Influences Tumor Escape from Immunologic Pressure

Human tumorigenesis is a multistep process that, similarly to chronic infection, may take place over several years [78]. Burnet and Thomas postulated the immunological resistance of the host against the development of cancer, terming it tumor immunosurveillance. Various research groups have reported data supporting the existence of antitumor immune responses, and the immunosurveillance hypothesis has led to the concept of immunoediting [79]. As remarked above, the first-described epigenetic changes in human cancer were losses of DNA methylation. It was apparent that metastases show more cancer-linked DNA hypomethylation than does the primary tumor. This would indicate that methylation deregulation begins early in carcinogenesis and would imply the acquisition of new advantages for the cancerous cells. The timing and progression of DNA methylation changes during carcinogenesis are not completely understood. It has been shown, however, that in an isogenic human mammary epithelial cell culture model of transformation, in order to acquire immortality and malignancy, cultured finite lifespan mammary cells must overcome two distinct proliferation barriers, and that in the transition from a finite lifespan to a malignantly transformed one, the aberrant DNA methylation changes occur in a stepwise fashion early in the transformation process [80]. Though DNA hypomethylation is a ubiquitous feature of carcinogenesis, regional hypermethylation can occur very early in tumorigenesis and it can also be strongly associated with tumor progression, thus serving as an indicator of survival [81].

The concept of cancer immunoediting has three phases: the first, elimination, refers essentially to cancer immunosurveillance, in which cells of the innate and adaptive immune response recognize and destroy developing tumors [82]. This phase takes place early in carcinogenesis, even before malignant transformation methylation deregulation is present, and therefore numerous aberrant methylation changes may be present within pre-malignant lesions. As remarked above, DNA hypomethylation is a ubiquitous feature of carcinogenesis and it can lead to the activation of genes with germline-specific expression. The MAGE-A1 promoter contains at least five activating regions, although activity appears to be driven mainly by two inverted ETS (Erytroblast Transformation Specific) motifs contained in regions B′ and B [83]. We have shown that DNA methylation of these regions appears to be the principal mechanism in the control of MAGE-A1 expression in the presence of ETS transcriptional factor. This epigenetic change at the start of carcinogenesis may lead to the ectopic expression of proteins and thus to the appearance of new antigens. At this level, therefore, tumor cells can be recognized by the immune system in a specific way, but the adaptive immunity depends on innate immunity. The double strand DNA is immunogenic and DNA hypomethylation increases its immunogenicity rendering it more reactive to innate immune system cells, inducing the maturation of dendritic cells [84]. DNA methylation has been related with some age-associated diseases, such as increases in autoimmune phenomena or chronic inflammation. Human DNA is an example of a self-antigen that undergoes age-associated genetic and epigenetic alterations [85]. In cancer, the apoptotic phenomenon and the necrosis of pre-tumor cells, cause a release of hypomethylated DNA, thus starting the immune response. The elimination phase might be seen as representing the beginning of the immune response against tumor cells.

The second phase, equilibrium, is a protracted period in which the tumor and the immune system enter a dynamic equilibrium [86]. At this level, the finite lifespan cells become malignantly transformed in a stepwise fashion. This second step coincides with immortalization, and results in hundreds of additional DNA methylation changes [80]. The early stage of carcinogenesis in breast tumors is characterized by the inactivation of cyclin-dependent kinase inhibitor P16 (gene CDKN2A), which is expressed at a high level in senescence arrest in human mammary epithelial cells [87]. Hypermethylation of CDKN2A has been documented in precancerous lesions and histologically normal breast tissues [88,89]. CpG islands in different gene promoters could be hypermethylated and therefore inactivated. In this group of genes, some could be involved in the immune response. As observed above, the expression of classical HLA class I may be modulated by methylation. In addition, the hypermethylation of IFN-regulatory factor 1 (IRF-1) gene has been found to bring about inhibition of IFN-gamma-mediated HLA class I expression in two melanoma cell lines [90]. Moreover, changes in HLA class I antigen expression may also reflect alterations in transcriptional regulation of the APM components. The latter play a critical role in the assembly and presentation of functional HLA class I antigens [60]. It has been shown that HDACi treatment can activate the proteasomal components (LMP2, LMP7), transporters associated with antigen processing (TAP1, TAP2) and the TAP-associated glycoprotein (tapasin) gene in tumor cells [91]. It has been confirmed that treatment with HDACi can enhance the surface expression of MHC class I in different cell lines [92,93]. HDAC-mediated chromatin repression has been reported as a major mechanism for the down regulation of class I transcription in tumor cells [94]. In this regard, and as remarked above, HDAC-mediated chromatin repression is directly related to DNA methylation. Cytotoxic T cells play a central role in the elimination of virally infected and tumor cells and require HLA class I expression on these cells to guide their attack [95]. Tumor escape in response to immune pressure has been observed in cancer patients enrolled in T-cell-based immunotherapy trials [96]. The MSR3-mel melanoma cell line was established from a metastatic lesion resected from a patient with melanoma who showed no response to vaccination with the MAGE-3.A1 peptide [97]. We have shown that the non expression of class I antigens in this cell line is due to the hypermethylation of these genes. Treatment with demethylating agent 5′-aza-2′-desoxycytidine (DAC) allowed HLA-A and -B transcription, restoring cell surface expression of HLA class I antigens and tumor cell recognition by MAGE-specific cytotoxic T lymphocytes [59]. It has also been reported that the antigen-specific recognition of cervical cancer cells by cytotoxic T lymphocytes is enhanced by treatment of the cancer cells with different histone deacetylase inhibitors, alone or in combination with DNA methylation inhibitors [98]. At this stage, the set of tumor cells may present heterogeneity in the expression of different proteins involved in the immune response.

In the third phase, escape, tumor variants that emerge from immune selection during the equilibrium phase develop into clinically apparent tumors that grow in immunocompetent hosts [99]. In this phase, tumor cells may escape the immune pressure in different ways. Tumor cells synthesize different immunomodulators that may prevent a direct attack from the immune system. Interleukine-10 is produced by Treg lymphocytes and is a modulator of immunoresponse. In this respect, methylation of the interleukin-10 gene in breast cancer tissues is lower than that in normal and benign breast tissues, and DNA hypomethylation in the gene influences gene activation. It has been proposed that hypomethylation of the IL-10 gene could be involved in the process of breast carcinogenesis [100]. In this respect, IL-10 has been related to the regulation of MICA expression in tumor cells. MICA binds to the NKG2D that is an activating receptor on NK cells, playing an important role in the cell-mediated immune response to tumors. It seems that IL-10 is a significant element in the modulation of NKG2D ligand (MICA), decreasing its expression; therefore, this interleukine may prevent the tumor cytotoxicity mediated by the NKG2D/MICA axis [101]. In addition, other proteins, not directly related to the immune response, may interfere, for example clusterin (CLU), a ubiquitous glycoprotein that plays an important role in many biological processes, one of which is complement regulation, preventing the insertion of the C5b65 complex into the cell membrane and thus protecting cells against immunological damage [102]. It has been reported that CLU expression tends to increase during cellular transformation and tumor progression [103,104]. We have shown that demethylation of the CLU promoter region provokes the reactivation of gene expression. Considering the potential role of extracellular CLU as an inhibitor of the complement (MAC insertion) as well as an inhibitor of apoptosis, it is tempting to speculate that the hypomethylated status of CLU results from the expansion of CLU-positive tumor cells selected because they are resistant to the immune pressure [105].

Finally, it should be taken into account that rate-limiting molecular processes for malignant proliferation, such as oncogene activation, may also trigger the loss of tumor recognition by the host's immune system. The relationship has been demonstrated between RAS/MAPK-dependent signal transduction and the hypermethylation of genes in HRAS-transformed fibroblasts [106]. The epigenetic silencing of the pro-apoptotic Fas gene in KRAS transformed cells has also been shown, suggesting that RAS-mediated signal transduction and DNMT-activity are closely linked in cancer [107]. Furthermore, it has been shown that APM component deficiencies occur more frequently in Ki-ras-mutated colorectal carcinoma lesions, and that they appear to be associated with the disease stage [108]. In this respect, the down-regulation of HLA class I and NKG2D ligands through the concerted action of Ras-MAPK (Ras-mitogen-activated protein kinase) and DNA methyltransferases in colorectal cancer cells has been reported. Cosuppression of HLA class I and NKG2D ligands and genes encoding APM mediate a strong functional link between Ras activation, DNMT activity and disruption of the antigen-presenting system controlling immune recognition in colorectal cancer cells. These results confirm the functional relevance of the joint action of DNA methylation and Ras/MAPK signalling in impairing efficient tumor cell recognition by the adaptative immune system. Although the molecular mechanism of down-regulation at the level of individual target genes remains unclear, it is likely that the two regulatory principles, DNA methylation and oncogenic signalling, exert a systemic effect on the HLA class I antigen-presenting system, because they also control the expression of peptide transporters (TAP1, TAP2, tapasin) and of proteosomal proteins (LMP2, LMP7) [109].

## 7. Demethylating and Deacetylating Agents as Anti-tumor Factors

Cancer is both a genetic and an epigenetic disease. However, unlike genetic changes, epigenetic ones can be reverted. Promoter hypermethylation can be targeted by inhibitors of DNMT, which may be nucleoside or non-nucleoside analogues. The most widely used and effective DNMT inhibitors include 5-azacytidine, 5-aza-2′deoxycytidine (5-aza-CdR), 5,6-dihydro-5-azacytidine and zabularine, as nucleoside analogues. Recently, nucleoside deoxycytidine analogues have received considerable attention, as a demethylating agent for the treatment of haematological malignancies [110]. However, such agents exert poor activity on solid tumors, presenting common adverse effects such as myelosuppression, severe gastrointestinal events and instability in aqueous solutions, thus limiting their application [111]. Non-nucleoside inhibitors of DNMT function without being incorporated into the DNA and so are theoretically less toxic than the nucleoside analogues. These DNMT inhibitors include procaine, mitoxatrone, N-acetyl-procainamide, procainamide and hydralazine. Despite the promising results obtained in preclinical trials, the applicability of non-nucleoside DNA methylation inhibitors to humans has met with only limited success. A recent study compared the activity of different nucleoside and non-nucleoside inhibitors of DNMT and found a functional diversity among them [112]. The conclusion reached was that 5-aza-CdR is far more effective in DNA methylation inhibition as well as in reactivating genes than are non-nucleoside inhibitors [113]. In addition to the question of nucleoside and non-nucleoside analogues, there has been increasing interest in the development of small molecules targeting DNMT. However, the DNMT family presents so many redundant functions that more than one member must be inhibited in order to optimally activate tumor-suppressor genes. A recent study suggested that MG98, an antisense compound to DNMT1, could partially down-regulate DNMT1, but no objective clinical response was observed [114]. When designing clinical trials involving demethylating agents, it should be borne in mind that the downstream effects on neoplastic behavior are diversified and even conflicting. These agents could have different effects depending on the pattern of genes methylated in a given tumor. Hypermethylation at a DNA promoter region does not necessarily lead to gene silencing. In some situations, DNA methylation may not be involved in epigenetic gene silencing, for example trimethylated H3K27, which has been shown to silence tumor suppressor genes, independent of promoter methylation [115].

The combined inhibition of DNA methylation and histone acetylation not only enhances gene re-expression but also drug sensitivity *in vivo*. In this respect, there is considerable interest in the potential use of epigenetic therapies in combination with existing chemotherapeutic agents, both for improving initial tumor response and for overcoming acquired drug resistance. It has been reported that treatment of ovarian and colon cell lines with 2-deoxy-5′azacytidine (decitabine, DAC) results in the partial reversal of DNA methylation, the re-expression of methylated loci such as Hmlh1 (important in determining sensitivity to chemotherapeutic agents) and sensitisation to cisplatin and carboplantin both *in vitro* and *in vivo* [116]. It has also been shown that the combination of the histone deacetylase inhibitor trichostatin A with decitabine is more effective in reactivating the transcription of epigenetically silenced genes in tumor cell lines than either drug alone [117].

How can this treatment affect the immune antitumor response? Defects in MHC class I surface expression on tumor cells may lead to the inability of the cytotoxic T cell to directly destroy tumor cells. The loss or dysfunction of molecules involved in antigen processing and presentation (such as TAP1, TAP2, LMP2, LMP7 and Tapasin) via the class I pathway contributes to deficient class I expression in several tumor types [118,119]. We have shown the effect of 2-deoxy-5′azacytidine on melanoma MSR3 cell line HLA class I expression and the restoration of the antigen-specific CTL response. Treatment with several demethylating agents may lead to the re-expression in tumor cells, of genes with germline-specific expression becoming tumor antigens. It has also been shown that 2-deoxy-5′azacytidine can up-regulate MAGE-A1 expression in different tumor cell lines [73], and the treatment of mice with decitabine induces the re-expression of MAGE-A1 in tumor cell line Xenografts [120]. It has also been reported that histone deacetylase inhibitor (HDACi)-treated tumor cells, including trichostatin A (TSA) and valproic acid, are capable of presenting antigens via the MHC class II pathway and also of enhancing the expression of molecules (TAP1, TAP2, LMP2, LMP7, Tapasin and MHC class I) involved in antigen processing and presentation via the MHC class I in melanoma cells. Other molecules that can be enhanced in their expression are the co-stimulatory molecules CD40 and CD86 (B7.2) [91]. In view of these findings, it can be concluded that treatment with DNA methylation inhibitors and HDACi, from the immunological point of view, can sensitize tumor cells to the immune response, and even turn them into antigen-presenting cells (APC). Nevertheless, and as mentioned above, the function of class II HLA molecules remains controversial with respect to tumor progression.

Despite the promising results of some clinical studies, these agents can seem paradoxical in anticancer therapies; many tumors are characterized by a global DNA hypomethylation, and some investigators have raised the question that the longer term use of demethylation could in itself be carcinogenic [121].

## 8. Conclusions

HLA expression by tumor cells plays a critical role in the generation of tumor antigen (TA)-specific immune response. Loss of these antigens can occur at the genetic, transcriptional and post-transcriptional levels. It is clear that both hyper- and hypomethylation of DNA are linked to carcinogenesis. Methylation modulates expression of HLA and associated molecules in malignant cells. In addition, many of the antigens present in the HLA context (cancer-germline genes) are the result of epigenetic modification. Therefore, epigenetics directly influences tumor escape from immunologic pressure. However, to date, demethylating agents seem to exert poor activity, presenting common adverse effects.
